# Measles Vaccination Supports Millennium Development Goal 4: Increasing Coverage and Increasing Child Survival in Northern Ghana, 1996–2012

**DOI:** 10.3389/fpubh.2018.00028

**Published:** 2018-02-12

**Authors:** Paul Welaga, Abraham Hodgson, Cornelius Debpuur, Peter Aaby, Fred Binka, Daniel Azongo, Abraham Oduro

**Affiliations:** ^1^Navrongo Health Research Centre, Navrongo, Ghana; ^2^OPEN, Institute of Clinical Research, University of Southern Denmark, Odense, Denmark; ^3^Research and Development Division, Ghana Health Service, Accra, Ghana; ^4^Bandim Health Project, Indepth Network, Bissau, Guinea-Bissau; ^5^Bandim Health Project, Statens Serum Institut, Copenhagen, Denmark; ^6^University of Health and Allied Sciences, Ho, Ghana

**Keywords:** measles vaccine, measles, vaccination, non-specific effects of vaccines, child survival

## Abstract

**Background:**

Measles vaccine (MV) administered as the last vaccine after the third dose of diphtheria-tetanus-pertussis (DTP) may be associated with better child survival unrelated to prevention of measles infection. Other studies have shown that MV administered after DTP was more beneficial and was associated with lower mortality compared with DTP administered after MV or DTP administered simultaneously with MV. We compared the difference in mortality between measles vaccinated after DTP3 and measles-unvaccinated children in Navrongo, Ghana.

**Methods:**

This was a follow-up study involving annual cohort of children aged 9–23 months from 1996 to 2012. We assessed survival in relation to the measles vaccination status within the first 12 months from interview date and until 5 years of age using Cox proportional hazards models.

**Results:**

In all, 38,333 children were included in the study. The proportion of children vaccinated with MV-after-DTP3 increased from 45% in 1996 to 95% in 2012. The adjusted hazard ratio (HR) for measles unvaccinated compared with MV-after-DTP3 vaccinated children was 1.38 (1.15–1.66) in the first 12 months after assessment of vaccination status and 1.22 (1.05–1.41) with follow-up to 5 years of age. The national immunization days campaigns with oral polio vaccine or MV might have reduced the effect of being MV-after-DTP3 vaccinated vs MV-unvaccinated. For 12 months of follow-up, the HR before a campaign for MV-unvaccinated children was 1.63 (1.23–2.17) compared to those who received MV-after-DTP3. After the campaign, the HR reduced to 1.23 (0.97–1.54). Stratifying the analysis by sex, measles-unvaccinated boys had a HR of 1.69 (1.33–2.61) compared to measles-unvaccinated girls who had a HR 1.06 (0.79–1.40) during 1-year follow-up. In 1989, only 7% of children in the area had received MV-after-DTP3; the increase in MV-after-DTP3 coverage from 1989 to 2012 may have lowered mortality rate among children aged 9 months to 3 years by 24%.

**Conclusion:**

Though an observational study, our findings suggest that measles vaccination, administered in the recommended sequence, is associated with improved child survival and may have contributed importantly to the mortality decline toward the achievement of Millennium Development Goal 4.

## Introduction

The introduction of the WHO’s Expanded Programme on Immunization (EPI) through a combination of routine and mass immunization programs against the six childhood killer diseases (tuberculosis, diphtheria, tetanus, pertussis, poliomyelitis, and measles) has contributed greatly toward reducing childhood mortality and morbidity (1). Much of the decline in childhood mortality in developing countries can be attributed to the impact of immunizations (2, 3).

The current vaccination schedule in Ghana requires each child to receive one dose of Bacille Calmette–Guerin (BCG) at birth, four doses of oral polio vaccine (OPV) (at birth, 6, 10, and 14 weeks), three doses of pentavalent (Penta) vaccine (at 6, 10, and 14 weeks), two doses of rotavirus at (6 and 10 weeks), three doses of pneumococcal vaccines (at 6, 10, and 14 weeks), two doses of measles (at 9 and 18 months), and one dose of yellow fever (at 9 months). Penta vaccine (diphtheria, tetanus, pertussis, *Haemophilus influenzae* type b, and hepatitis B) was introduced in January 2002 to replace diphtheria–tetanus–pertussis (DTP), and in May 2012, three additional vaccines; pneumococcal, rotavirus, and second dose measles vaccine (MV) at 18 months were added to the vaccination schedule.

Evaluations of immunization programs are usually based on the assumption that vaccines have an impact only against the specific disease. Each vaccine is assessed based on its ability to prevent the occurrence of a particular disease or prevent children from dying from that disease (4). The estimated effect of these vaccines on childhood mortality may be underestimated, particularly for MV (2, 3, 5–13). When MV was introduced in Africa in the late 1970s and early 1980s, several studies found major reductions in childhood mortality (14, 15). Population based studies in Asia and the Americas reported similar results. The 30–86% reduction on childhood mortality from MV reported in these studies was much larger than anticipated, as measles infection accounted for less than 30% of the deaths in these communities (3). The reductions found in childhood mortality are in addition to those associated with measles infection. Hence, these observations suggest that MV may have non-specific beneficial effects (NSEs) that provide protection against infections other than measles (3).

The NSEs of vaccines might be explained by cross reactive T- and B-cell epitopes (heterologous immunity) or trained innate immunity or both (16–18). Recent studies of BCG indicate that the NSEs may be due to epigenetic changes reprogramming innate immunity (“immune training”) to a stronger pro-inflammatory response also to unrelated pathogens and may change once the child gets vaccinated with a new vaccine (19, 20).

The WHO’s Strategic Advisory Group of Experts on Immunization (SAGE) recently conducted a thorough review of the potential NSEs of vaccines and concluded in the epidemiological review that MV may be associated with beneficial non-specific effects (6). In the immunological review, SAGE concluded that the findings neither excluded nor confirmed the possibility of beneficial or deleterious non-specific immunological effects of the vaccines under study (BCG, DTP, MV) on all-cause mortality (21).

The WHO and other international organizations have been advocating for improved vaccination coverage levels. Coverage for measles vaccination in Ghana by the end of 2014 was 89% (22). What is often not emphasized is the timeliness of administering the vaccines and the strict adherence to the recommended sequence of vaccinations. Previous studies in Africa and Asia have suggested that administering DTP simultaneously with MV or DTP after MV may be associated with higher mortality than having MV as the most recent vaccination (7–11, 13, 23).

We have previously analyzed the effect of getting MV-after-DTP3 compared with getting DTP with or after MV, i.e., MV-out-of-sequence, which showed that receiving DTP with MV or DTP after MV was associated with 42% higher mortality than having MV as the most recent vaccination, i.e., MV-after-DTP3 (13). Hence, this study showed how much has been gained in child survival by increasingly following the recommended schedule. In this study, we have focused on examining the difference in mortality between having received MV-after-DTP3 and being MV-unvaccinated. We, further, assessed whether the effect differed between males and females and whether the effect was affected by mass vaccination campaigns with OPV or MV. Finally, we examined to what extent increasing the proportion of children receiving MV-after-DTP3 might have contributed to the decline in child mortality toward the achievement of Millennium Development Goal 4.

## Materials and Methods

### Study Setting

The study area is the Kassena-Nankana East and West Districts in the Upper East region of northern Ghana (24, 25). The estimated population is 160,000 under continuous demographic surveillance with about 8% (4,000) of the current population aged 9–23 months. It covers a land area of 1,675 km^2^. It has a hospital that serves as a referral hospital to seven health centers, a private clinic and over 40 Community Health Compounds located in rural communities and manned by trained nurses who provide basic health care as well as routine vaccinations. The area is mostly rural (80%) with primary occupation being subsistence agriculture. During the period of the study, under-five mortality declined from 235/1,000 in 1996 to 51.5/1,000 deaths in 2012 (Figure S1 in Supplementary Material). The estimated population of resident children aged 9 to 23 months in the study area during the period of the study from 1996 to 2012 was about 60,000.

### Vaccination Data

Routine vaccination data from the Navrongo Health and Demographic Surveillance system (HDSS) were used for this study. From 1996 to 2010, vaccination data were collected once annually from October to December by fieldworkers from health cards of children less than 2 years of age, except in 2001 when the HDSS failed to collect vaccination data (13). From 2011 to 2012, vaccination data were collected every 4 months for children below 3 years of age.

The HDSS field teams visit all households three to four times a year to document demographic events, educational status, and household possession. The demographic events routinely collected include births, deaths, pregnancies, marriages, and migrations. All residents in the surveillance area are assigned a unique ID, and all demographic events, including the records of the dead, are linked using each individual’s unique ID. The HDSS is a monitoring tool for assessing the impact of health interventions (24, 26).

Before the Navrongo HDSS was established, vaccination data were also collected from health cards in 1989–1991 during a vitamin A trial for children aged 6 months to 5 years (26, 27). In this trial, the children were followed every 4 months for a period of 2 years to distribute vitamin A supplements and to ascertain survival.

### Ethics Approval

This study was carried out in accordance with the recommendations of the Institutional Review Board of the Navrongo Health Research Centre. The study area has a HDSS that is used to monitor the population dynamics of the communities. Being a HDSS site that required routine frequent visits (about three to four times in a year) to households to update health and demographic events such as births, deaths, in-migrants and out-migrants, we sought verbal consent from heads of households for the routine collection of the demographic events. We also collected and updated routine vaccination data of children below 3 years during the three to four visits in a year to households. For the vaccination data collection, we sought verbal consent from the mother or father or, in the absence of the parents, any adult caregiver to document the routine vaccines the child has received from the health card. The consent process was approved by the institutional review board of the Navrongo Health Research Centre.

### Statistical Methods

Data were entered into a database system developed in Visual FoxPro 6.0 and analyzed using STATA 12.1. The vaccination records of each child were linked to their death records from the HDSS database using their unique IDs to ascertain their survival status. The analysis was limited to children aged 9–23 months at the time of assessment of vaccination status as this is the age group relevant for MV; the children were only included the first time if their vaccination card was seen more than once in this age group. The MV-vaccinated children were those who received MV after the last DTP dose (DTP3). The MV-unvaccinated children included those who did not receive any vaccine at all, or received other vaccines including DTP but not MV. Survival analysis using Cox proportional hazards models with age as the underlying time and reported as mortality rate ratios (MRRs) with 95% confidence interval were used to assess the association between measles vaccinations status at enrollment and subsequent mortality. The proportional hazards assumption for the Cox proportional hazard models were examined (Figure S2 in Supplementary Material). We also adjusted for socioeconomic status (wealth index), sex, maternal education, and interview year. A wealth index was computed from household assets using principal component analysis. Mortality was assessed prospectively from the interview date using the landmark approach to reduce survival bias for 12 months of follow-up and then till they attained age 5 years. Follow-up was censored on 30th April 2012 because two vaccines, rotavirus and pneumococcal vaccines, and an additional MV at 18 months were introduced in May 2012 into Ghana’s immunization program. Analysis was stratified by the periods DTP (1996–2001) and Penta (2002–2012) were used and by sex. In analyzing the data collected during the vitamin A trial in 1989–1990, we adjusted for age, ownership of radio, zone, and weight for age.

The specific effects of vaccines in providing immunity against targeted infections are likely to be maintained over several years. However, the non-specific effects may be due to epigenetic changes reprogramming innate immunity and may change once the child gets a new vaccination (28, 29). It is, therefore, likely the national immunization days (NID) campaigns with OPV or MV could have changed the vaccination status because these vaccines are likely to become the most recent vaccination for the children after the campaign. We examined this possibility by calculating the mortality rates and hazard ratios (HR) before and after the campaign. This was treated as an intention-to-treat analysis assuming that all children got the campaign vaccine. The analysis included all deaths including the very few deaths from measles. National coverage data suggest that participation was very high and individual level coverage data from the study area from 2011 to 2012 indicate that campaigns reached at least 86–94% of the eligible population (13).

A Community-based Health Planning and Services (CHPS) was tested in Navrongo in the period 1994–2003 where trained nurses were relocated into communities to provide basic health care and routine vaccinations services. During the experiment, the study area was divided into four experimental cells. Cell1 had community health volunteers (CHVs) only, Cell2 had trained community health nurses called community health officers (CHOs) only, Cell3 had both CHOs and CHVs, and Cell4 was the comparison cell with no interventions. We examined how improved routine vaccination services in the villages with nurses affected measles after DTP3 vaccination coverage during the trial period.

We also censored for measles deaths in some of our analyses to assess to what extent the changes in mortality were due to the prevention of measles infection. Measles deaths were identified using verbal autopsy procedures. We interviewed the mother or guardian using verbal autopsy tools to ascertain the circumstances leading to the death of the child. The VA instruments contain a set of questions as well as a section for verbatim narrations of the circumstances leading to the death. Three physicians each reviewed the verbal autopsy forms and assigned an underlying cause of death. If at least two agreed on the underlying cause of death to be measles, a diagnosis was established.

## Results

**Figure 1:**
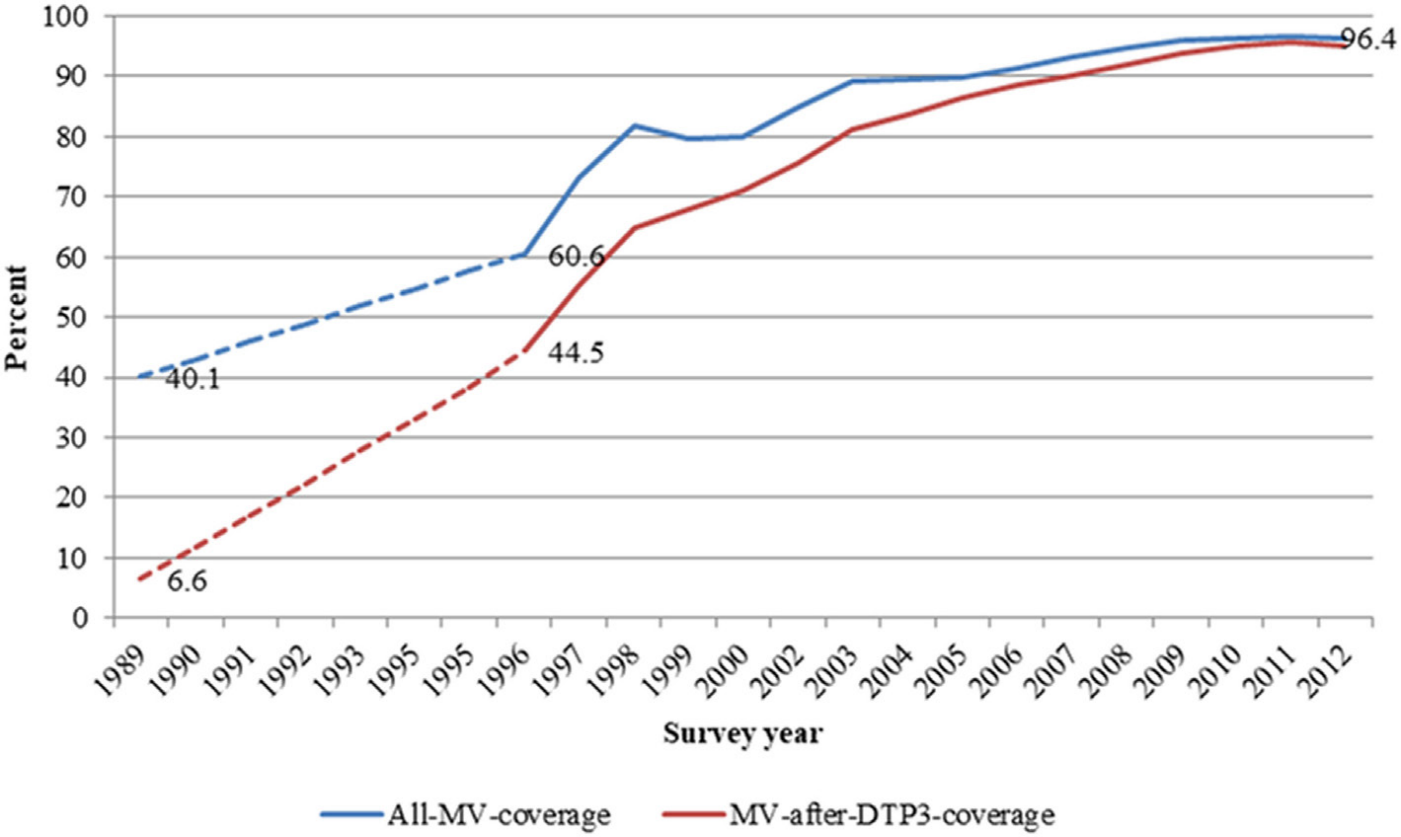
Trends in all measles and measles after DTP3 vaccination coverages among children aged 12–23 months in Navrongo Health and Demographic Surveillance system: 1989–2012.

In all, 38,333 children aged 9–23 months and enrolled between 1996 and 2012 were included in the analysis. About 50.5% (19,354) were males. The proportion of children vaccinated with MV-after-DTP3 increased from 44.5% (1,169/2,627) in 1996 to 95.5% (3,813/3,994) in 2011. Overall, measles vaccination coverage increased from 60.6 (1,591/2,627) to 96.7% (3,863/3,994) in the same period. In 1989–1990, about 40% of children aged 9–23 months were vaccinated against measles, and just 6.6% were vaccinated with measles after DTP3 (Figure 1). Table 1 compares the background characteristics of children included in the study, i.e., the MV-after-DTP3 and no-MV groups. The distribution of the children by sex, mother’s education, wealth index, age at enrollment, and interview year vary slightly within the MV-after-DTP3 and no-MV groups (Table 1).

**Table 1:** Comparison of the background characteristics of children included in the study by vaccination status.

Variable	Measles vaccine (MV)-after-DTP3 *n*(%)	No MV *n*(%)	*P*-value
**Sex**			
Male	16,585 (50)	2,769 (52)	0.046
Female	16,398 (50)	2,581 (48)	

**Mother’s education**			
No education	13,286 (40)	2,819 (53)	<0.001
Primary/JSS	15,856 (48)	2,054 (38)	
Secondary/tertiary	2,902 (9)	246 (5)	
Missing	939 (3)	231 (4)	

**Wealth index**			
Poorest	7,820 (24)	1,359 (25)	<0.001
Poorer	6,364 (19)	1,167 (22)	
Poor	6,185 (19)	1,105 (21)	
Less poor	6,734 (20)	992 (19)	
Least poor	4,813 (15)	576 (11)	
Missing	1,067 (3)	151 (3)	

**Child’s age**			
9–11	1,906 (6)	1,104 (21)	<0.001
12–14	9,819 (30)	1,466 (27)	
15–17	7,801 (24)	1,059 (20)	
18–20	7,285 (22)	1,055 (20)	
21–23	6,172 (19)	666 (12)	

**Interview year**			
1996	1,169 (4)	1,036 (19)	<0.001
1997	1,201 (4)	585 (11)	
1998	1,561 (5)	439 (8)	
1999	1,733 (5)	519 (10)	
2000	1,956 (6)	551 (10)	
2002	2,057 (6)	409 (8)	
2003	2,298 (7)	306 (6)	
2004	2,442 (7)	310 (6)	
2005	2,461 (7)	289 (5)	
2006	2,347 (7)	234 (4)	
2007	2,350 (7)	176 (3)	
2008	2,676 (8)	158 (3)	
2009	2,529 (8)	113 (2)	
2010	1,357 (4)	55 (1)	
2011	3,813 (12)	131 (2)	
2012	1,033 (3)	39 (1)	

Total	32,983 (86.0)	5,350 (14.0)	

### Mortality during Follow-up

Children not vaccinated with MV at enrollment had 38% higher risk of dying than those vaccinated with MV-after-DTP3 during 12 months of follow-up [HR = 1.38 (1.15–1.66)] (Table 2). When mortality was assessed from interview date until 5 years of age, measles-unvaccinated children had 22% higher risk of dying than children vaccinated with MV-after-DTP3 [HR = 1.22 (1.05–1.41)]. The size of the beneficial effect of MV did not change when we censored for measles deaths (Table S1 in Supplementary Material).

**Table 2:** Mortality rate and rate ratios (MRR) for children aged 9–23 months comparing children vaccinated with measles after DTP3 and measles-unvaccinated children at time of survey and by period.

Period	Mortality rate per 1,000 person-years (deaths/person-years)		MRR No-Measles vaccine (MV) vs MV (age and year adjusted)	MRR No-MV vs MV (Adjusted)	% with MV	Measles death
	Received MV-after-DTP3	No MV				
**Mortality within 12 months of follow-up**						
1989–1990	20 (2/102)	72 (64/892)	3.77 (0.93–15.36)	3.44 (0.84–14.07)^a^	10	13
1996–2012	18 (520/29,611)	39 (190/4,827)	1.43 (1.19–1.71)	1.38 (1.15–1.66)^b^	86	4
1996–2001 [diphtheria-tetanus-pertussis (DTP) era]	25 (233/9,412)	42 (135/3,243)	1.34 (1.08–1.68)	1.30 (1.04–1.62)^b^	74	4
2002–2011 [Pentavalent (Penta) era]	14 (287/20,200)	35 (55/1,584)	1.56 (1.15–2.12)	1.52 (1.12–2.05)^b^	93	0

**Mortality up to 5 years of follow-up**						
1996–2012	10 (875/87,562)	17 (276/16,001)	1.26 (1.09–1.45)	1.22 (1.05–1.41)^b^	86	7
1996–2001 (DTP era)	13 (407/31,353)	18 (194/10,989)	1.14 (0.95–1.36)	1.10 (0.92–1.32)^b^	74	7
2002–2012 (Penta era)	8 (468/56,209)	16 (82/5,011)	1.48 (1.16–1.89)	1.44 (1.13–1.84)^b^	93	0

*^a^Adjusted for age, ownership of radio, zone, and weight for age*.

*^b^Adjusted for age, socioeconomic status (wealth index), sex, maternal education, and interview year*.

Stratifying the analysis by periods in which DTP and Penta were used, children without MV in the DTP period (1996–2001) had 30% higher risk of dying in the first 12 months of follow-up [HR = 1.30 (1.04–1.62)] than those who received MV-after-DTP3. In the Penta period (2002–2012), children without MV had 52% higher risk of dying during 12 months of follow-up than those who received MV after Penta3 [HR = 1.52 (1.12–2.05)] (Table 2). The risk associated with not receiving MV did not change when we censored for measles deaths (Table S1 in Supplementary Material). When the children were followed from the assessment date until they were 5 years old, children without MV during the DTP period had 10% higher risk of dying than those with MV after DTP3 [HR = 1.10 (0.92–1.32)]. In the Penta period, children without MV had 44% higher risk of dying than those who received MV after Penta3 [HR = 1.44 (1.13–1.84)] when they were followed from the assessment date until they were 5 years old. The risk of dying for MV-unvaccinated children was higher during the Penta period compared to the DTP period in the first 12 months after assessment of vaccination status (52 vs 30%) (*P* = 0.021), and with follow-up to 5 years of age (44 vs 10%) (*P* = 0.005).

We, further, classified the MV-unvaccinated children into two groups; children who received other vaccines but not MV, and those who did not receive any vaccine (unvaccinated). The adjusted HR for those who received other vaccines except MV compared with MV-after-DTP3 vaccinated children was 1.42 (1.18–1.72) in the first 12 months after assessment of vaccination status and 1.24 (1.06–1.44) with follow-up to 5 years of age (Table S2 in Supplementary Material). For the unvaccinated, the adjusted HR compared with MV-after-DTP3 vaccinated children was 1.14 (0.75–1.73) in the first 12 months after assessment of vaccination status and 1.10 (0.78–1.56) with follow-up to 5 years of age (Table S2 in Supplementary Material).

### Measles Vaccination Status at Enrollment and Subsequent Mortality by Sex

We assessed the effects of being MV-after-DTP3 vaccinated on subsequent mortality separately for males and females. Being measles vaccinated was associated with lower mortality only among boys; measles-unvaccinated boys had 69% higher risk during 1 year of follow-up [HR = 1.69 (1.33–2.61)] and 43% higher mortality [HR = 1.43 (1.18–1.73)] when followed until 5 years of age (Table 3). For girls, the HR = 1.06 (0.79–1.40) during 1 year of follow-up, and 0.98 (0.78–1.24) when followed until 5 years of age. The effect differed significantly for boys and girls (*P* = 0.001; test of homogeneity).

**Table 3:** Measles vaccination status at enrollment and subsequent mortality by sex.

Variable	Mortality within 12 months of follow-up		Mortality up to 5 years of follow-up	
	Mortality rate per 1,000 pyrs	Adjusted HR^a^	Mortality rate per 1,000 pyrs	Adjusted HR^a^
Vaccination status at enrollment	–			

**Males**				
Measles vaccine (MV)-after-DTP3	16.8 (250/14,902)	Ref	10.0 (441/44,134)	Ref
No MV	48.1 (120/2,497)	1.69 (1.33–2.61)	20.7 (171/8,271)	1.43 (1.18–1.73)
**Females**				
MV-after-DTP3	18.4 (270/14,709)	Ref	10.0 (434/43,428)	Ref
No MV	30.0 (70/2,330)	1.06 (0.79–1.40)	13.6 (105/7,729)	0.98 (0.78–1.24)

*^a^Adjusted for age, socioeconomic status (wealth index), maternal education, sex, and interview year*.

### Measles Vaccination Status and Subsequent Mortality before and after OPV or Measles Campaigns

To examine whether national OPV or measles campaigns affected the estimated effect of receiving MV-after-DTP3 on subsequent mortality, we calculated the mortality rates and HR before and after any campaign during follow-up. For 12 months of follow-up, the HR before a campaign for MV-unvaccinated children was 1.63 (1.23–2.17) compared to those who received MV-after-DTP3. After the campaign, the HR reduced to 1.23 (0.97–1.54) (Table 4). If we limited the analysis to MV campaigns, the HR before a campaign was 1.41 (1.17–1.70) for MV-unvaccinated children but reduced to 0.92 (0.40–2.09) after the campaign. Similar mortality patterns were observed before and after campaigns during the DTP and Penta periods, and among females (Table 4). The campaigns may have reduced the effect of being MV-after-DTP3 vaccinated vs MV-unvaccinated.

**Table 4:** Mortality rates and hazard ratios (HR) before and after an oral polio vaccine (OPV) or Measles campaign for children aged 9–23 months by vaccination status in Navrongo, Ghana: 1996–2012.

Variable	Mortality before OPV or measles vaccine (MV) campaign from interview date		Mortality after OPV or MV campaign	
	Mortality rate per 1,000 pyrs	Adjusted HR^a^	Mortality rate per 1,000 pyrs	Adjusted HR^a^
**Vaccination status at enrollment**	All children			
MV-after-DTP3	19 (144/7,719)	Ref	17 (376/21,892)	Ref
No MV	52 (86/1,653)	1.63 (1.23–2.17)	33 (104/3,174)	1.23 (0.97–1.54)

**Children delivered between 1994 and 2001 [diphtheria-tetanus-pertussis (DTP) period]**				
MV-after-DTP3	26 (80/3,111)	Ref	24 (153/6,301)	Ref
No MV	54 (72/1,337)	1.60 (1.15–2.22)	33 (63/1,906)	1.09 (0.80–1.47)

**Children delivered between 2002 and 2011 [Pentavalent (Penta) period]**				
MV-after-DTP3	14 (64/4,608)	Ref	14 (223/1,5,592)	Ref
No MV	44 (14/316)	1.84 (0.98–3.42)	32 (41/1,268)	1.40 (0.98–2.00)

**Males**				
MV-after-DTP3	20 (77/3,852)	Ref	16 (173/11,049)	Ref
No MV	61 (51/840)	1.69 (1.16–2.46)	42 (69/1,658)	1.68 (1.24–2.26)

**Females**				
MV-after-DTP3	17 (67/3,866)	Ref	19 (203/10,843)	Ref
No MV	43 (35/813)	1.52 (0.98–2.36)	23 (35/1,516)	0.81 (0.56–1.18)

*^a^Adjusted for age, socioeconomic status (wealth index), maternal education, sex, and interview year*.

### CHPS Impact on Measles Vaccination Coverage

The proportion of MV-after-DTP3 vaccinated children was higher in the villages with CHOs [RR = 1.29 (1.23–1.35)] than the areas with no intervention (Cell4) (Table 5). In the non-intervention area, measles-unvaccinated children had 78% higher mortality [HR = 1.78 (1.25–2.52)] than those vaccinated. Similarly, measles-unvaccinated children had 38% higher mortality in the intervention cells than those who received MV [HR = 1.38 (0.99–1.94)] (Table 5). The data we used to compare the effect of MV in areas with community nurses and control areas with no intervention was limited to the period of the intervention from 1996 to 2003. Even though the relative risk of dying for MV-unvaccinated children was higher in the non-intervention communities than the communities with nurses (78 vs 38%), this difference was not statistically significant (*P* = 0.557). The reduced risk of dying for MV-unvaccinated children in the intervention areas compared with those in the non-intervention areas could be a result of the intensive child centered activities of the nurses and other health care volunteers in the intervention areas during the period of the intervention. These estimates are probably conservative because many children got MV during follow-up, with a higher proportion of children in the intervention cells (72%) receiving MV compared to those in the non-intervention cells (52%). The intervention with CHOs improved child survival and became a national health delivery policy in Ghana (24, 25).

**Table 5:** Relative risk and mortality rate ratio (MRR) for measles vaccination after DTP3 vs No MV, comparing villages with community nurses and control villages with no intervention.

	Community nurses (Cell2 + 3)	No intervention (Cell4)	Relative risk
**Vaccination status at enrollment**			
No of children aged 9–23 months	8,884	4,475	
Received measles vaccine (MV) after DTP3	81% (7,237/8,884)	63% (2,817/4,475)	1.29 (1.23–1.35)
No MV	19% (1,647/8,884)	37% (1,658/4,475)	0.50 (0.47–0.54)

**Mortality during follow-up (1-year)**	**Mortality rate/1,000 pyrs (deaths/person-years)**	**Mortality rate/1,000 pyrs (deaths/person-years)**	

Received MV after DTP3	22.5 (155/6,889)	25.3 (66/2,609)	0.89 (0.66–1.21)
No MV	34.6 (53/1,532)	47.4 (71/1,498)	0.73 (0.50–1.06)
MRR No-MV vs MV^a^ (adjusted)	1.38 (0.99–1.94)	1.78 (1.25–2.52)	
Proportion receiving MV during follow-up for children with No MV during first visit	72.1% (75/104)	51.5% (53/103)	1.40 (0.97–2.03)

*Children interviewed between 1996 and 2003*.

*^a^Adjusted for age, sex, socioeconomic status (wealth index), maternal education, and interview year*.

## Discussion

### Main Observations

In this study, we found a significant reduction in mortality of 38% during 1 year of follow-up after assessment of vaccination status and 22% with follow-up to 5 years of age for MV-after-DTP3 vaccinated children compared to measles-unvaccinated children. We observed similar beneficial patterns of receiving MV-after-DTP3 when we stratified the analysis by males and females (Table 3). The protective effect of MV-after-DTP3 vaccination on mortality was unchanged when we censored for measles deaths in our analysis. The reduction in mortality was particularly strong during the penta period from 2002 to 2012. Even though childhood mortality declined during the Penta period, the beneficial effect of MV did not change. It even appeared to be more beneficial at the Penta period when a lot more antigens were used compared to the DTP period. The mortality patterns before and after OPV or measles campaigns suggest that these campaigns may have reduced the effect of being MV-after-DTP3 vaccinated vs MV-unvaccinated. There have been major improvements in the coverage of MV-after-DTP3 vaccination in the study area, and this is likely to have contributed importantly to the mortality reduction toward achieving Millennium Development Goal 4 (MDG4) (Figure S1 in Supplementary Material).

### Strengths and Weaknesses

There are a number of weaknesses to this study. First, the children classified as MV-unvaccinated may have received MV during the follow-up period, particularly for those that were relatively young. About 59 and 67% of the MV-unvaccinated children contacted in subsequent visits in the first 12 months of follow-up and up to 5 years of age, respectively, had subsequently received MV. Only 7 and 13% of the MV-unvaccinated children were met in the first 12 months of follow-up and up to 5 years, respectively. Second, almost every year since 1996, there were national campaigns with either OPV or MV or both. These campaigns often occurred in October and November more or less at the same time that the annual survey of vaccination status was conducted and vaccination status assessed. Both of these weaknesses would tend to minimize the difference in mortality between MV-after-DTP3 and MV-unvaccinated and may explain why the estimates declined when the children were followed to 5 years of age. In other words, our estimates are conservative.

Being an observational study, there are likely other confounding factors that this study could not control for directly, such as general improvement in the health-care delivery system, health literacy as well as infrastructure, which could have affected the mortality rates and also increased MV coverage. One could also argue that the comparison group of no MV may represent the population with no access or difficult access to vaccines or health care, hence have higher mortality in general. However, differential access to health-care services is unlikely to explain all the excess mortality associated with MV-unvaccinated, as mortality was still much higher among MV-unvaccinated children compared with MV-after-DTP3 in both communities with nurses and control communities without nurses (Table 5). Furthermore, after general MV campaigns, the difference between the two groups disappeared making it unlikely that the difference in mortality is primarily due to inherent weaknesses or frailty.

This study also has several important methodological strengths. This study used so far the largest data set on routine vaccination from a low/middle-income country (13). It also made use of well-documented longitudinal data collected by the Navrongo HDSS which enabled us to track and monitor the survival status of the children and to control for important potential confounders and major determinants of child mortality in the study area.

The coverage for MV-after-DTP3 increased from 7% in 1989 to 95% in 2012. This change in MV-after-DTP3 coverage might have contributed importantly to the reduction in child mortality and the achievement of MDG4 in the study area. The improvement in MV-after-DTP3 vaccination coverage is related to a strong emphasis on routine services and can partly be attributed to improved health care delivery in the area, particularly with the introduction of the CHPS in which trained community health nurses are relocated into communities to provide basic health care as well as routine vaccinations. The community-based intervention trial took place in the study area and later led to the current Ghanaian policy on community-based health service delivery (24). As indicated in Table 5, this program increased the MV-after-DTP3 coverage considerably.

### Consistency and Contradiction with Previous Observations

Our results point to a beneficial effect of MV on child survival. The 38% higher mortality for MV-unvaccinated children would correspond to 28% lower mortality for MV-vaccinated children. Considering that this estimate is probably conservative, this is consistent with other studies from developing countries which have reported mortality reductions in the range of 30–86% (3, 6). Other studies comparing mortality rates before and after the introduction of MV reported major reductions in mortality after the introduction of MV (3, 28–31). The data from the present study also supported that MV may have non-specific beneficial effects which go beyond protecting children from dying from measles infection.

Measles vaccination was associated with beneficial effect for both boys and girls before receiving any campaign vaccine (Table 4). However, after the campaigns, we found a significantly stronger beneficial effect of measles vaccination for boys, but not for girls. It is unclear why the beneficial effect is stronger for boys as many other studies reported stronger beneficial effects of measles vaccination for girls than boys. For example the SAGE review suggested that MV had a 40% (22–53%) stronger effect for girls than boys (6). However, some studies have suggested that OPV is associated with stronger beneficial effects for boys than for girls (32, 33). Hence, we recommend that future studies examine the sex-differential effect of measles vaccination on all-cause mortality before and after OPV or measles campaigns.

### Interpretation

Generally, vaccines are assessed based on the immunogenicity of the vaccine or the ability of the vaccine to protect children from being infected or dying from the specific disease. However, the effect of MV in the present study was not explained by prevention of measles infection since the estimates changed little when measles deaths were censored in the survival analyzes. There are several reasons why our finding of higher mortality among MV-unvaccinated children is unlikely to be due to confounding or differential access to health care between MV-vaccinated and MV-unvaccinated children. First, we controlled for potential confounding factors such as age, sex, maternal education, interview year, and socioeconomic status, which may be associated with mortality and receiving MV. Second, differential access to care seems unlikely to explain a large part of the enigma since we showed similar tendencies for MV-unvaccinated vs MV-after-DTP3 in villages with and without nurses. The differential effect of MV was somewhat less in the villages with community nurses but unvaccinated children in these villages were also much more likely to be vaccinated with MV during follow-up; hence, the estimate is likely to be more conservative in villages with community nurses than in control villages. Third, if the higher mortality of MV-unvaccinated children was mainly due to inherent weaknesses among the unvaccinated children, then as more and more of the unvaccinated received MV, the difference between the groups should have disappeared. This did not happen. Fourth, if inherent weakness or frailty were the main reason for the difference in mortality between the MV-unvaccinated and the MV-after-DTP3 groups, then the HR (MV-unvaccinated/MV-after-DTP3) should have been the same before and after other vaccination campaigns. That was not the case; instead, as predicted, the difference declined after all children were likely to have received a live vaccine (OPV, MV) as their last vaccination. Importantly, after general MV campaigns, the mortality difference disappeared completely. It seems more logical to assume that the MV-after-DTP3 group had an immunological benefit compared with the MV-unvaccinated and not that the MV-unvaccinated group had high mortality due to inherent weaknesses or frailty. Fifth, since the HR for MV-after-DTP3 was stronger when compared with children who received other vaccines but not MV than with totally unvaccinated children, the beneficial effect of MV-after-DTP3 is likely to be due to MV and not because of other vaccines received in the routine vaccination programme. Sixth, randomized trials of MV have shown that MV as the most recent vaccine is associated with lower mortality which is not explained by prevention of measles infections (5, 34). It is, therefore, biologically plausible that MV-after-DTP3 may induce an immunological benefit compared with being MV-unvaccinated.

Previous studies have suggested that OPV might have beneficial NSEs (32, 35, 36). Findings from a randomized trial suggested that OPV might have beneficial non-specific effects that reduced all-cause mortality by 17% (30). Though the underlying biological mechanisms have not been fully studied, some studies suggest that OPV, just like BCG, is capable of inducing strong immune training (36). In this study, the NID campaigns with OPV or MV might have reduced the effect of being MV-after-DTP3 vaccinated vs MV-unvaccinated.

As has been shown for BCG, beneficial NSEs may be due to epigenetic changes reprogramming innate immunity. This may change once the child gets a new vaccination (19, 20). The specific immunological mechanisms behind the non-specific effects of vaccine are still being investigated, and it might be a mixture of heterologous immunity, trained innate immunity, and other types of changes in the immune system (16, 19).

It has recently been suggested that measles infection leads to immunosuppression which leads to excess non-measles mortality over the next 2–3 years and that the introduction of MV prevents this immune amnesia and, therefore, led to a more general reduction in non-measles mortality (37). However, this hypothesis was not supported by individual level data showing higher long-term mortality after measles infection. The studies which have examined long-term mortality have tended to find lower subsequent mortality if the individual survived the acute phase of measles infection (38, 39). Hence, it may not be the prevention of immune amnesia which explains the beneficial effect of MV. In Navrongo, there was very little measles for the last 10 years of the study period (Table 2), but having MV-after-DTP still had a major beneficial effect on child survival.

In a recent SAGE review, the studies of BCG and MV suggested almost a halving of mortality which could not be explained by the prevention of the targeted diseases (6). However, the non-specific effects of vaccines on overall mortality are yet to be considered in vaccine policy formulation. This study shows that MV-after-DTP3 vaccination is associated with considerable non-specific beneficial effect on child survival.

### Measles Vaccinations Contribution to the Decline in Child Mortality

Considering that children not vaccinated with MV are associated with 38% higher risk of dying than those who received MV-after-DTP3 during 1-year follow-up (Table 2), the increase in the proportion of children vaccinated with MV-after-DTP3 from 53 to 96.4% between 1996 and 2012 (Table S3 in Supplementary Material) would have meant a 14% reduction in childhood mortality for children aged 9 months to 3 years. This would amount to about 813 children likely to be saved by measles vaccination from an estimated 3,387 children aged 9–36 months that died between 1996 and 2012 in the area.

Going back to the vaccination data collected during the vitamin A trial in Navrongo from 1989 and 1991 when 10% had received MV-after-DTP3 to the current level of 96.4% in 2012 (Table S4 in Supplementary Material), the increase in coverage levels for MV-after-DTP3 vaccination would explain a 24% reduction in mortality for children aged 9 months to 3 years assuming not-being-MV-vaccinated to be associated with a 38% higher mortality than MV-after-DTP3 (Table S4 in Supplementary Material). The 24% reduction in mortality would translate into 1,285 children likely to be saved by measles vaccination from an estimated 5,352 children aged 9–36 months that died between 1990 and 2012 in the study area.

### Implications and Conclusion

This study shows that MV-after-DTP3 is clearly associated with considerable reductions in mortality even after censoring for measles deaths in the analysis. This has several implications. Our findings suggest that increasing the MV-after-DTP3 coverage has the potential to reduce child mortality in a major way. Considering the number of lives saved through measles vaccination, this might be a major contributor to achieving the MDG4 in the study area. In the current understanding of the decline in child mortality, MV is believed to have had a rather modest effect, particularly in the last decade. In Niger, for example, MV was only estimated to have produced 5% of the reduction in mortality between 1998 and 2009 (40). The process we have described for the last 25 years in Navrongo is likely to have occurred also in other low-income countries. Getting MV-after-DTP3 improves child survival irrespective of the timing of the measles vaccination. Some studies suggest that if MV is given early after DTP3, this may further reduce child mortality (12).

When Ghana launched its EPI in June 1978 with six antigens; BCG, measles, DTP, and oral polio vaccines were often delivered in campaigns with irregular intervals to reach certain targets (41) rather than through routine vaccination services. Vaccines were, therefore, often delivered out of sequence. Getting the vaccination program to function in a regular fashion may have had a much larger impact on child survival than usually assumed.

The WHO through EPI/SAGE should implement or encourage policies that would promote measles vaccination. The use of measles vaccination coverage as one of the monitoring targets may be needed to put stronger emphasis on measles vaccination. This would encourage vaccination program implementers to not only focus on DTP3 coverage, but also ensure that children get vaccinated with MV-after-DTP3 because of its survival benefits. Some studies suggest that live vaccines may have beneficial effects while inactivated vaccines may be associated with negative effects on child survival (6, 42). We should consider pursuing a *measles-vaccine-last policy* by ensuring that MV is the most recent vaccination through childhood. It is important to test the effect of MV and other routine vaccines on all-cause mortality in randomized trials.

Measles infection may be eliminated in the near future, and measles vaccination may be de-emphasized. We should continue to vaccinate with MV because of its beneficial effect in improving child survival.

## Author Contributions

PW and PA proposed the present analysis. All authors help supervise data collection. PW conducted the analysis and wrote the first draft of the paper. All authors contributed with comments and approved the final version of the paper.

## Conflict of Interest Statement

The authors declare that the research was conducted in the absence of any commercial or financial relationships that could be construed as a potential conflict of interest.

## Supplementary Material

Figure S1 Trends in under-five mortality in Navrongo HDSS: 1996–2011.

Figure S2 Checking for Cox proportional hazard assumptions.

## References

[1] Clemens J Holmgren J Kaufmann SH Mantovani A. Ten years of the global alliance for vaccines and immunization: challenges and progress. Nat Immunol (2010) 11:1069–72. 10.1038/ni1210-1069 21079627

[2] Kristensen I Aaby P Jensen H. Routine vaccinations and child survival: follow up study in Guinea-Bissau, West Africa. BMJ (2000) 321:1435–8. 10.1046/j.1467-0658.2001.0120e.x 11110734 PMC27544

[3] Aaby P Samb B Simondon F Seck AMC Knudsen K Whittle H Non-specific beneficial effect of measles immunisation: analysis of mortality studies from developing countries. Br Med J (1995) 311:481–5. 10.1136/bmj.311.7003.481 7647643 PMC2550544

[4] Aaby P Kollmann T Benn CS Non-specific effects of neonatal and infant vaccination – public health, immunological, and conceptual challenges. Nat Immunol (2014) 15:895–9. 10.1038/ni.2961 25232810

[5] Aaby P Martins CL Garly ML Bale C Anderson A Rodriques A Non-specific effects of standard measles vaccine at 4.5 and 9 months of age on childhood mortality: randomised controlled trial. BMJ (2010) 341:c6495. 10.1136/bmj.c6495 21118875 PMC2994348

[6] Higgins JPT Soares-Weiser K López-López JA Kakourou A Chaplin K Christensen H Association of BCG, DTP, and measles containing vaccines with childhood mortality: systematic review. BMJ (2016) 355:i5170. 10.1136/bmj.i5170 27737834 PMC5063034

[7] Aaby P Vessari H Nielsen J Maleta K Benn CS Jensen H Sex differential effects of routine immunizations and childhood survival in rural Malawi. Pediatr Infect Dis J (2006) 25:721–7. 10.1097/01.inf.0000227829.64686.ae 16874172

[8] Aaby P Ibrahim S Libman M Jensen H. The sequence of vaccinations and increased female mortality after high-titre measles vaccine: trials from rural Sudan and Kinshasa. Vaccine (2006) 24:2764–71. 10.1016/j.vaccine.2006.01.004 16457909

[9] Aaby P Jensen H Walraven G Age-specific changes in female-male mortality ratio related to the pattern of vaccinations: an observational study from rural Gambia. Vaccine (2006) 24:4701–8. 10.1016/j.vaccine.2006.03.038 16621182

[10] Aaby P Nielsen J Benn CS Trape JF Sex-differential and non-specific effects of routine vaccinations in a rural area with low vaccination coverage: observational study from Senegal. Trans R Soc Trop Med Hyg (2015) 109:77–84. 10.1093/trstmh/tru186 25573112

[11] Hirve S Bavdekar A Juvekar S Benn CS Nielson J Aaby P. Non-specific and sex-differential effects of vaccinations on child survival in rural western India. Vaccine (2012) 30:7300–8. 10.1016/j.vaccine.2012.09.035 23022401

[12] Aaby P Martins CL Ravn H Rodrigues A Whittle HC Benn CS Is early measles vaccination better than later measles vaccination? A review. Trans R Soc Trop Med Hyg (2015) 109:16–28. 10.1093/trstmh/tru174 25573106

[13] Welaga P Oduro A Debpuur C Aaby P Ravn H Binka F Fewer out-of-sequence vaccination and reduction of child mortality in Northern Ghana. Vaccine (2017) 35:2496–503. 10.1016/j.vaccine.2017.03.004 28341115

[14] Clemens JD Stanton BF Chakraborty J Chowdhury S Rao MR Ali M Measles vaccination and childhood mortality in rural Bangladesh. Am J Epidemiol (1988) 128:1330–9. 10.1093/oxfordjournals.aje.a115086 3195571

[15] Aaby P Bukh J Lisse IM Smits AJ. Measles vaccination and reduction in child mortality: a community study from Guinea-Bissau. J Infect (1984) 8:13–21. 10.1016/S0163-4453(84)93192-X 6699411

[16] Flanagan KL van Crevel R Curtis N Shann F Levy O Optimmunize Network Heterologous (“nonspecific”) and sex-differential effects of vaccines: epidemiology, clinical trials, and emerging immunologic mechanisms. Clin Infect Dis (2013) 57:283–9. 10.1093/cid/cit209 23572484 PMC3689344

[17] Benn CS Netea MG Selin LK Aaby P A small jab – a big effect: nonspecific immunomodulation by vaccines. Trends Immunol (2013) 34:431–9. 10.1016/j.it.2013.04.004 23680130

[18] Welsh RM Che J Brehm MA Selin LK. Heterologous immunity between viruses. Immunol Rev (2010) 235:244–66. 10.1111/j.0105-2896.2010.00897.x 20536568 PMC2917921

[19] Kleinnijenhuis J Quintin J Preijers F Joosten LA Saeed S Jacobs C Bacille Calmette-Guerin induces NOD2-dependent nonspecific protection from reinfection via epigenetic reprogramming of monocytes. Proc Natl Acad Sci U S A (2012) 109:17537–42. 10.1073/pnas.1202870109 22988082 PMC3491454

[20] Kleinnijenhuis J Quintin J Preijers F Benn CS Joosten LA Jacobs C Long-lasting effects of BCG vaccination on both heterologous Th1/Th17 responses and innate trained immunity. J Innate Immun (2014) 6:152–8. 10.1159/000355628 24192057 PMC3944069

[21] WHO. Weekly Epidemiologic Record (WER). (Vol. 89). (2014). p. 221–36. Available from: http://www.who.int/wer/2014/wer8921/en/

[22] Ghana Statistical Service (GSS), Ghana Health Service (GHS), ICF International. Ghana Demographic and Health Survey 2014. Rockville, MD, USA: GSS, GHS, and ICF International (2015).

[23] Aaby P Jensen H Samb B Cisse B Sodemann M Jakobsen M Differences in female-male mortality after high-titre measles vaccine and association with subsequent vaccination with diphtheria-tetanus-pertussis and inactivated poliovirus: re-analysis of West African studies. Lancet (2003) 361:2183–8. 10.1016/S0140-6736(03)13771-3 12842371

[24] Binka FN Bawah AA Phillips JF Hodgson A Adjuik M MacLeod B. Rapid achievement of the child survival millennium development goal: evidence from the Navrongo experiment in Northern Ghana. Trop Med Int Health (2007) 12:578–83. 10.1111/j.1365-3156.2007.01826.x 17445125

[25] Nyonator FK Koku JAW Phillips JF Jones TC Miller RA. The Ghana community-based health planning and services initiative for scaling up service delivery innovation. Health Policy Plan (2005) 20(1):25–34. 10.1093/heapol/czi003 15689427

[26] Ross D Dollimore N Smith PG Kirkwood BR Arthur P Morris SS Vitamin A supplementation in northern Ghana: effects on clinic attendances, hospital admissions, and child mortality. Ghana VAST study team. Lancet (1993) 342(8862):7–12. 10.1016/0140-6736(93)91879-Q 8100345

[27] Welaga P Nielsen J Adjuik M Debpuur C Ross DA Ravn H Non-specific effects of diphtheria-tetanus-pertussis and measles vaccinations? An analysis of surveillance data from Navrongo, Ghana. Trop Med Int Health (2012) 17:1492–505. 10.1111/j.1365-3156.2012.03093.x 23006334

[28] Aaby P Bhuyia A Nahar L Knudsen K de Francisco A Strong M The survival benefit of measles immunization may not be explained entirely by the prevention of measles disease: a community study from rural Bangladesh. Int J Epidemiol (2003) 32:106–15. 10.1093/ije/dyg005 12690020

[29] The Kasongo Project Team Balen H Mercenier P Daveloose P Bruycker M Grosdene P Influence of measles vaccination on survival pattern of 7-35-month-old children in Kasongo, Zaire. The Kasongo project team. Lancet (1981) 317(8223):764–7. 10.1016/S0140-6736(81)92634-9 6110963

[30] Desgrées du Loû A Pison G Aaby P. Role of immunizations in the recent decline in childhood mortality and the changes in the female/male mortality ratio in rural Senegal. Am J Epidemiol (1995) 142:643–52. 10.1093/oxfordjournals.aje.a117688 7653475

[31] Aaby P Samb B Simondon F Knudsen K Coll Seck AM Bennett J Divergent mortality for male and female recipients of low–titer and high–titer measles vaccines in rural Senegal. Am J Epidemiol (1993) 138:746–55. 10.1093/oxfordjournals.aje.a116912 8237989

[32] Lund N Andersen A Hansen ASK Jepsen FS Barbosa A Biering-Sørensen S The effect of oral polio vaccine at birth on infant mortality: a randomized trial. Clin Infect Dis (2015) 61:1504–11. 10.1093/cid/civ617 26219694 PMC4614411

[33] Benn CS Jacobsen LH Fisker AB Rodrigues A Sartono E Lund N Campaigns with oral polio vaccine may lower mortality and create unexpected results. Vaccine (2017) 35:1113–6. 10.1016/j.vaccine.2016.11.006 28139347 PMC5312669

[34] Aaby P Garly ML Balé C Martins C Jensen H Lisse IM Survival of previously measles-vaccinated and measles-unvaccinated children in an emergency situation: an unplanned study. Pediatr Infect Dis J (2003) 22(9):798–805. 10.1097/01.inf.0000083821.33187.b5 14506371

[35] Aaby P Hedegaard K Sodemann M Nhante E Veirum JE Jakobsen M Childhood mortality after oral polio immunisation campaign in Guinea-Bissau. Vaccine (2005) 23:1746–51. 10.1016/j.vaccine.2004.02.054 15705481

[36] Aaby A Andersen A Martins CL Fisker AB Rodrigues A Whittle H Does oral polio vaccine have non-specific effects on all-cause mortality? Natural experiments within a randomised controlled trial of early measles vaccine. BMJ Open (2016) 6:e013335. 10.1136/bmjopen-2016-013335 28011813 PMC5223718

[37] Mina MJ Metcalf CJ De Swart RL Osterhaus AD Grenfell BT. Long-term measles-induced immunomodulation increases overall childhood infectious disease mortality. Science (2015) 348(6235):694–9. 10.1126/science.aaa3662 25954009 PMC4823017

[38] Aaby P Samb B Andersen M Simondon F. No long-term excess mortality after measles infection: a community study from Senegal. Am J Epidemiol (1996) 143:1035–41. 10.1093/oxfordjournals.aje.a008667 8629610

[39] Aaby P Samb B Simondon F Cisse B Jensen H Lisse IM Low mortality after mild measles infection compared to uninfected children in rural west Africa. Vaccine (2002) 21:120–6. 10.1016/S0264-410X(02)00430-9 12443670

[40] Amouzou A Habi O Bensaïd K Niger countdown case study working group. Reduction in child mortality in Niger: a countdown to 2015 country case study. Lancet (2012) 380(9848):1169–78. 10.1016/S0140-6736(12)61376-2 22999428

[41] Ministry Of Health/Ghana Health Service. Immunization Programme Comprehensive Multi-Year Plan (2010-2014). Available from: http://www.nationalplanningcycles.org/sites/default/files/country_docs/Ghana/revised_cmyp_2010_-_2014.pdf (assessed: 01/02/2018)

[42] Aaby P Benn CS Nielsen J Lisse IM Rodriques A Ravn H. Testing the hypothesis that diphtheria-tetanus-pertussis vaccine has negative non-specific and sex-differential effects on child survival in high-mortality countries. BMJ Open (2012) 2:e000707. 10.1136/bmjopen-2011-000707 22619263 PMC3364456

