# Supplementary material for: Measles Vaccination Supports Millennium Development Goal 4: Increasing Coverage and Increasing Child Survival in Northern Ghana, 1996–2012
Source: Front Public Health. 2018 Feb 12;6:28. doi: 10.3389/fpubh.2018.00028 (PMC5816587; doi:10.3389/fpubh.2018.00028)
Supplement: Supplementary file 1 [file Table_1.DOCX]

**Supplementary table 1: Mortality rate and rate ratios (MRR) for children aged 9-23 comparing children vaccinated with measles after DTP3 and measles unvaccinated children at time of survey and by period censoring for measles deaths**

| **Mortality within 12 months of follow-up censoring for measles deaths** | | | | | | | |
| --- | --- | --- | --- | --- | --- | --- | --- |
| Period | Mortality rate per 1000 person-years (deaths/person-years) | | MRR No-MV vs MV (age and year adjusted) | | MRR No-MV vs MV^#^  ( Adjusted) | % with MV | Measles death |
|  | Received MV after DTP3 | No MV |  |  |  |  |  |
| 1989-1990 | 20 (2/102) | 58 (51/885) | 2.38 (0.58-9.82) | | 2.13 (0.51-8.90)* | 10% | 13 |
| 1996-2012 | 18 (520/29610) | 39 (186/4824) | 1.41 (1.17-1.69) | | 1.36 (1.13-1.64) | 86% | 4 |
| 1996-2001  (DTP era) | 25 (233/9411) | 40 (131/3240) | 1.31 (1.05-1.64) | | 1.27 (1.01-1.59) | 74% | 4 |
| 2002-2011  (Penta era) | 14 (287/20200) | 35 (55/1584) | 1.56 (1.15-2.12) | | 1.52 (1.12-2.05) | 93% | 0 |
| **Mortality up to five years of follow-up censoring for measles death** | | | | | | | |
| Period | Mortality rate per 1000 person-years (deaths/person-years) | | | MRR No-MV vs MV (age and year adjusted) | MRR No-MV vs MV^#^  (Adjusted) | % with MV | Measles death |
|  | Received MV after DTP3 | No MV | |  |  |  |  |
| 1996-2012 | 10 (874/87559) | 17 (270/15997) | | 1.24 (1.07-1.43) | 1.20 (1.04-1.39) | 86% | 7 |
| 1996-2001  (DTP era) | 13 (406/31350) | 17 (188/10986) | | 1.11 (0.93 -1.33) | 1.08 (0.90-1.29) | 74% | 7 |
| 2002-2011  (Penta era) | 8 (468/56209) | 16 (82/5011) | | 1.48 (1.16-1.89) | 1.44 (1.13-1.84) | 93% | 0 |

^*^Adjusted for age, ownership of radio, zone and weight for age

^#^ adjusted for age, socioeconomic status (wealth index), maternal education, sex and interview year
