# Supplementary material for: Measles Vaccination Supports Millennium Development Goal 4: Increasing Coverage and Increasing Child Survival in Northern Ghana, 1996–2012
Source: Front Public Health. 2018 Feb 12;6:28. doi: 10.3389/fpubh.2018.00028 (PMC5816587; doi:10.3389/fpubh.2018.00028)
Supplement: Supplementary file 2 [file Table_2.DOCX]

**Supplementary Table 2: Mortality rate and rate ratios (MRR) for children aged 9-23 months comparing children vaccinated with measles after DTP3 with measles unvaccinated children at time of survey and by period**

| **Mortality within 12 months of follow-up** | | | |
| --- | --- | --- | --- |
| **Vaccination status** | **Mortality rate per 1000 pyrs** | **MRR (age adjusted)** | **MRR^#^ (adjusted** |
| **Period: 1996-2012** |  |  |  |
| MV-after-DTP3 | 18 (520/29611) | ref | ref |
| Other vaccines, no MV | 39 (160/4077) | 1.47 (1.22-1.78) | 1.42 (1.18-1.72) |
| Unvaccinated | 40 (30/749) | 1.19 (0.78-1.80) | 1.14 (0.75-1.73) |
| **DTP Era: 1996-2001** |  |  |  |
| MV-after-DTP3 | 25 (233/9412) | ref | ref |
| Other vaccines, no MV | 41 (108/2604) | 1.39 (1.10-1.75) | 1.34 (1.06-1.69) |
| Unvaccinated | 42 (27/639) | 1.15 (0.73-1.80) | 1.10 (0.70-1.73) |
| **Penta Era: 1996-2001** |  |  |  |
| MV-after-DTP3 | 14 (287/20200) | ref | ref |
| Other vaccines, no MV | 35 (52/1473) | 1.59 (1.16-2.16) | 1.54 (1.13-2.10) |
| Unvaccinated | 27 (3/111) | 1.27 (0.40-3.99) | 1.21 (0.39-3.81) |
| **Mortality up to five years of age** | | | |
| **Vaccination status** | **Mortality rate per 1000 pyrs** | MRR (age adjusted) | MRR (adjusted |
| **Period: 1996-2012** |  |  |  |
| MV-after-DTP3 | 10 (875/87562) | ref | ref |
| Other vaccines, no MV | 17 (232/13567) | 1.28 (1.1-1.49) | 1.24 (1.06-1.44) |
| Unvaccinated | 18 (44/2434) | 1.14 (0.81-1.60) | 1.10 (0.78-1.56) |
| **DTP Era: 1996-2001** |  |  |  |
| MV-after-DTP3 | 13 (407/31353) | ref | ref |
| Other vaccines, no MV | 18 (156/8912) | 1.16 (0.96-1.40) | 1.12 (0.93-1.35) |
| Unvaccinated | 18 (38/2078) | 1.04 (0.72-1.52) | 1.02 (0.7-1.48) |
| **Penta Era: 2002-2011** |  |  |  |
| MV-after-DTP3 | 8 (468/56209) | ref | ref |
| Other vaccines, no MV | 16 (76/4655) | 1.48 (1.15-1.90) | 1.44 (1.12-1.85) |
| Unvaccinated | 17 (6/356) | 1.59 (0.71-3.58) | 1.51 (0.67-3.39) |

^#^ adjusted for age, socioeconomic status (wealth index), sex, maternal education and interview year

MRR - Mortality rate ratio
