# Supplementary material for: Measles Vaccination Supports Millennium Development Goal 4: Increasing Coverage and Increasing Child Survival in Northern Ghana, 1996–2012
Source: Front Public Health. 2018 Feb 12;6:28. doi: 10.3389/fpubh.2018.00028 (PMC5816587; doi:10.3389/fpubh.2018.00028)
Supplement: Supplementary file 3 [file Table_3.DOCX]

**Supplementary table 3: Calculation of the decline in mortality due to measles vaccination using only mortality during 12 months of follow-up**

| 1996-2012 | Overall mortality | | |
| --- | --- | --- | --- |
| **1996** | Proportion vaccinated | Relative Mortality rate | Total mortality |
| MV-after-DTP3 | 0.53 | 1 | 0.53 |
| No MV | 0.47 | 1.38 | 0.6486 |
|  |  |  | 1.1786 |
| **2012** |  |  |  |
| MV-after-DTP3 | 0.964 | 1 | 0.964 |
| No MV | 0.036 | 1.38 | 0.04968 |
|  |  |  | 1.01368 |
| **Change in mortality**  **1996-2012** |  |  | 1.01368/1.1786 = 0.8601 =>14% reduction |
|  |  |  |  |
| **1989** |  |  |  |
| MV-after-DTP3 | 0.10 | 1 | 0.1 |
| No MV | 0.90 | 1.38 | 1.242 |
|  |  |  | 1.342 |
| **Change in mortality**  **1989-2012** |  |  | 1.01368/1.342 = .7554 =>24% reduction |
