# Supplementary material for: Measles Vaccination Supports Millennium Development Goal 4: Increasing Coverage and Increasing Child Survival in Northern Ghana, 1996–2012
Source: Front Public Health. 2018 Feb 12;6:28. doi: 10.3389/fpubh.2018.00028 (PMC5816587; doi:10.3389/fpubh.2018.00028)
Supplement: Supplementary file 4 [file Table_4.DOCX]

**Supplementary table 4: Vaccination coverage for MV-after-DTP3**

| Year | MV-OOS | MV-after-DTP3 | no MV | All | Percentage coverage for MV-after-DTP3/(MV-after-DTP3+no MV) |
| --- | --- | --- | --- | --- | --- |
| 1989-1991 | 504 | 104 | 941 | 1549 | 10 |
| **DTP period**  1996 | 371 | 1169 | 1,036 | 2576 | 53 |
| 1997 | 352 | 1201 | 585 | 2138 | 67.2 |
| 1998 | 368 | 1561 | 439 | 2368 | 78.1 |
| 1999 | 266 | 1733 | 519 | 2518 | 77 |
| 2000 | 197 | 1956 | 551 | 2704 | 78 |
| **Penta period**  2002 | 217 | 2057 | 409 | 2683 | 83.4 |
| 2003 | 180 | 2298 | 306 | 2784 | 88.2 |
| 2004 | 150 | 2442 | 310 | 2902 | 88.7 |
| 2005 | 82 | 2461 | 289 | 2832 | 89.5 |
| 2006 | 61 | 2347 | 234 | 2642 | 90.9 |
| 2007 | 68 | 2350 | 176 | 2594 | 93 |
| 2008 | 63 | 2676 | 158 | 2897 | 94.4 |
| 2009 | 43 | 2529 | 113 | 2685 | 95.7 |
| 2010 | 13 | 1357 | 55 | 1425 | 96.1 |
| 2011 | 40 | 3813 | 131 | 3984 | 96.7 |
| 2012 | 10 | 1033 | 39 | 1082 | 96.4 |

Note: MV-OOS: Measles vaccination Out-of-sequence (i.e. receiving MV with DTP or DTP after MV)
