# Supplementary figures and images for: Measles Vaccination Supports Millennium Development Goal 4: Increasing Coverage and Increasing Child Survival in Northern Ghana, 1996–2012
Source: Front Public Health. 2018 Feb 12;6:28. doi: 10.3389/fpubh.2018.00028 (PMC5816587; doi:10.3389/fpubh.2018.00028)

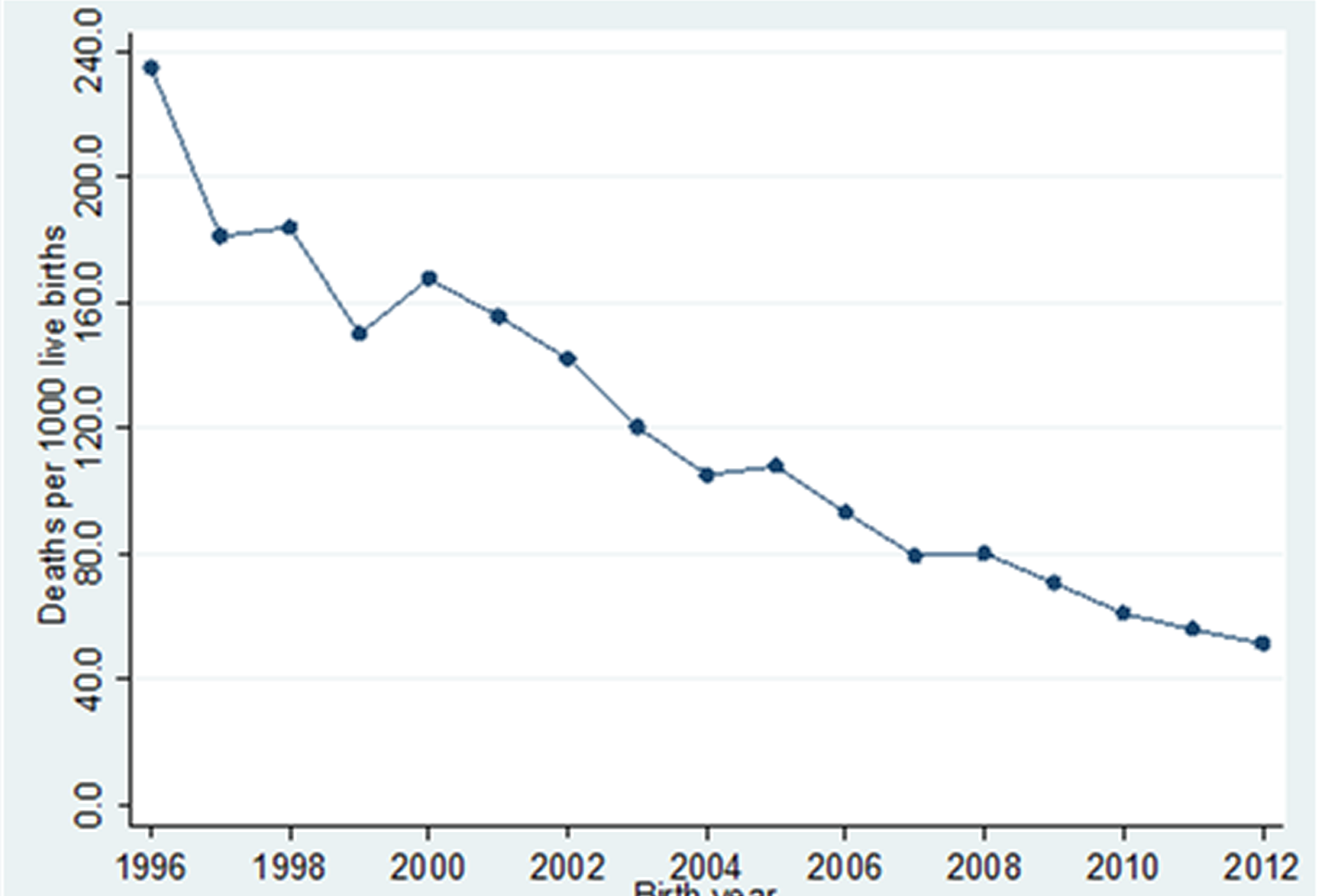

Supplement: Figure S1 — Trends in under-five mortality in Navrongo HDSS: 1996–2011. [file Image_1.TIF]

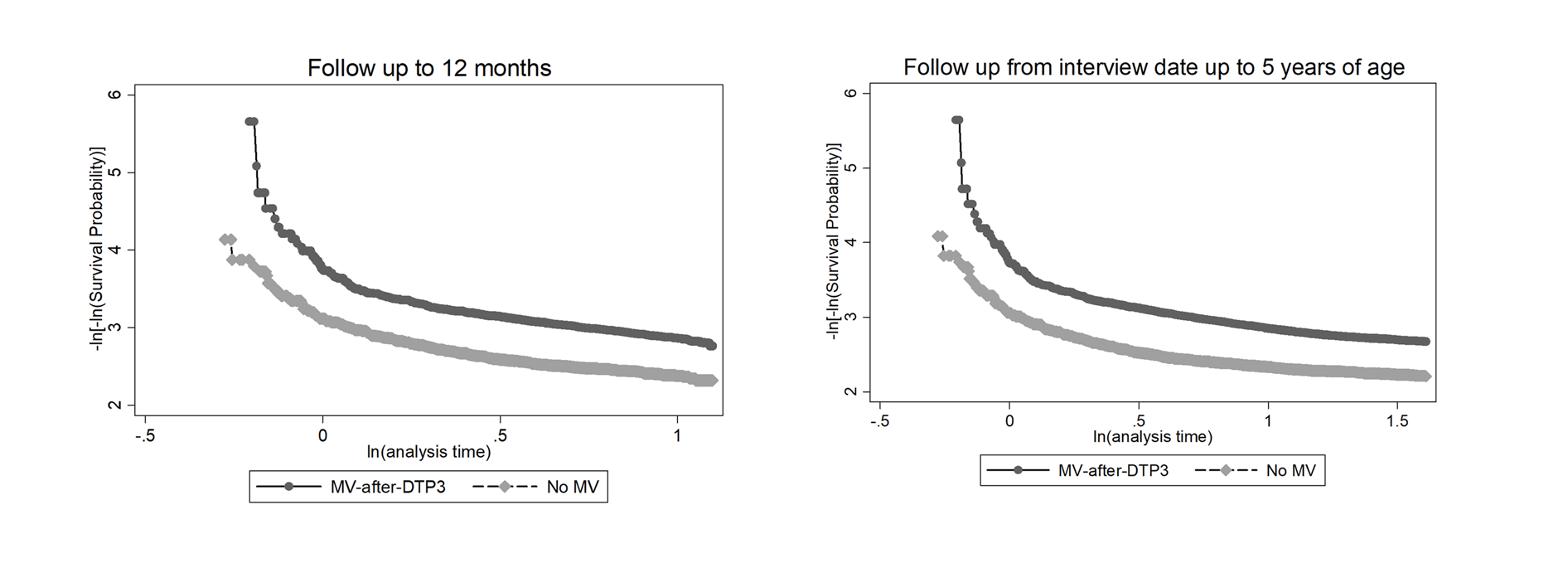

Supplement: Figure S2 — Checking for Cox proportional hazard assumptions. [file Image_2.TIF]
